# Improving Depth Resolution of Ultrasonic Phased Array Imaging to Inspect Aerospace Composite Structures †

**DOI:** 10.3390/s20020559

**Published:** 2020-01-20

**Authors:** Reza Mohammadkhani, Luca Zanotti Fragonara, Janardhan Padiyar M., Ivan Petrunin, João Raposo, Antonios Tsourdos, Iain Gray

**Affiliations:** School of Aerospace, Transport and Manufacturing (SATM), Cranfield University, Cranfield MK43 0AL, UK; l.zanottifragonara@cranfield.ac.uk (L.Z.F.); m.padiyar@cranfield.ac.uk (J.P.M.); i.petrunin@cranfield.ac.uk (I.P.); j.raposo@cranfield.ac.uk (J.R.); i.gray@cranfield.ac.uk (I.G.)

**Keywords:** ultrasonic NDE, autonomous inspection, ultrasonic phased array, NDT, composite materials, depth resolution, defect sizing

## Abstract

In this paper, we present challenges and achievements in development and use of a compact ultrasonic Phased Array (PA) module with signal processing and imaging technology for autonomous non-destructive evaluation of composite aerospace structures. We analyse two different sets of ultrasonic scan data, acquired from 5 MHz and 10 MHz PA transducers. Although higher frequency transducers promise higher axial (depth) resolution in PA imaging, we face several signal processing challenges to detect defects in composite specimens at 10 MHz. One of the challenges is the presence of multiple echoes at the boundary of the composite layers called structural noise. Here, we propose a wavelet transform-based algorithm that is able to detect and characterize defects (depth, size, and shape in 3D plots). This algorithm uses a smart thresholding technique based on the extracted statistical mean and standard deviation of the structural noise. Finally, we use the proposed algorithm to detect and characterize defects in a standard calibration specimen and validate the results by comparing to the designed depth information.

## 1. Introduction

This work is part of the EU-H2020 FET-OPEN CompInnova project [1] that aims to develop an innovative solution for the automatic Non-Destructive Testing (NDT) inspection, sizing, localization and repair of damages on aircraft composite structures [2,3]. For the NDT inspection phase, the CompInnova solution employs two different and complementary technologies: (i) Infrared Thermography (IRT) to detect near-surface defects, and (ii) ultrasonic Phased Array (PA) for sub-surface defects. The combination of these two methods in the overlapping areas is also considered as the future work in CompInnova, in order to improve the accuracy of detection. These modules are mounted on a vortex robot for autonomous inspection of composite structures [4].

There is an increasing use of ultrasonic phased array [5,6,7,8,9,10,11,12] in recent years in comparison to conventional single transducer ultrasonic inspections for NDT, due to their flexibility, speed of operation and good imaging performance. Ultrasonic phased arrays are able to capture multiple A-scans (full waveform) and provide a B-scan (a cross-sectional view of the specimen to show depth and size information of defects) at each measurement [5,6]. The authors in [6] showed that ultrasonic wheel arrays can produce C-scans (a top-view of the material to illustrate location and size of defects) with a comparable quality to an immersion system, with a much shorter scanning time. A recent study has compared the ultrasonic inspection of composite materials using single element and phased array ultrasonic testing methods [13]. This study highlighted that, although both methods can be used for inspecting materials with a thickness of up to 25 mm, the phased array provides more stable signal parameters and a higher chance of detection for lower signal strength.

The use of autonomous systems for non-destructive testing utilising a wide range of sensing techniques has been explored by several researchers in the literature. For instance, robotic platforms have been designed and developed for the inspections of long welded lines [14]. A relatively recent literature review about climbing robots was carried out by Schmidt et al. [15]. The use of unmanned aerial systems for the visual inspection of aircraft wing panels was described by Malandrakis et al. [16]. The application of ultrasonic phased array for automated inspections has been recently explored in [17,18], leveraging different technologies such as robotic manipulators and robots for in-pipe inspections [19,20]. The solution proposed in the CompInnova project aims to step change NDT inspections during scheduled maintenance C-check and D-check (heavy maintenance checks), allowing faster repeatable inspections and accurate localization, sizing and classification of defects. Having these maintenance checks automated, we can achieve a significant reduction in both costs and inspection time [3]. A vortex-based robotic platform [21] is the solution chosen for the PA and IRT automation of scanning process.

However, when the inspected material is not homogeneous, conventional ultrasonic imaging techniques are not effective, and we need to employ signal processing techniques to improve the temporal resolution of the ultrasonic signal [22]. Some studies suggest to use a combination of Wiener filtering and autoregressive (AR) spectral extrapolation to improve signal-to-noise ratio (SNR) and temporal (axial) resolution of the ultrasonic inspection [23,24,25]. They use Wiener filter for deconvolution, then, using a part of the deconvolved spectrum with high SNR, an AR model of the process is built. Next, the rest of the spectrum is extrapolated by the obtained AR model. However, performance of this method strongly depends on the width of the frequency window and order of the AR filter [23,25].

Some other works assume that back-scattered ultrasonic echoes can be modeled as superimposition of multiple Gaussian echoes and try to estimate multiple unknown parameters of such model from the received signals [26,27]. This solution needs a multi-dimensional signal optimization over multiple parameters, which requires extra processing power and may not converge to the optimal solution [28].

Wavelet analysis is another method that has been widely used in many signal processing applications for signal estimation, classification, compression and de-noising. [29]. Due to its multi-resolution characteristics for signal decomposition, it has attracted significant attention from many researchers in the area of ultrasonic NDT [30,31,32,33,34,35,36].

### 1.1. Challenges of Automated Post-Processing of PA Data

In general, phased arrays are able to electronically control their beam to scan, steer or sweep their beams. One of the main advantages of the PA inspection with full waveform data capture and storage is the ability to extract the depth information using sliding gate (window) analysis. Depth information is crucial in the assessment of the defect, the state of the component and to decide whether repair, replacement or no-action is needed. In addition, for the CompInnova concept, the depth information is very valuable because it allows an accurate calculation of the volume and area of material that the laser will remove during repair process.

**Figure 1:**
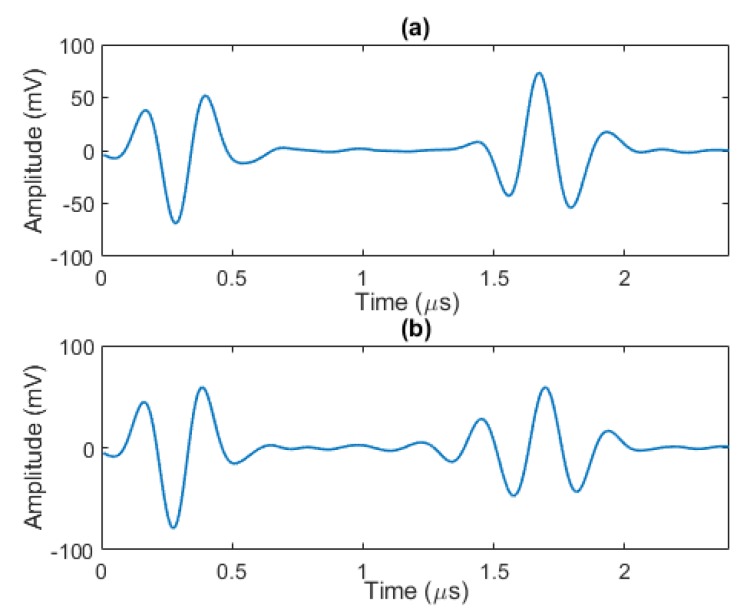
Two different A-scans using 5 MHz PA transducer: (**a**) at a non-defective location, and (**b**) at a location with defect close to back wall.

Dead zone and limited axial (depth) resolution are the main drawbacks related to the use of lower frequency PA ultrasonic transducers for defect detection and characterization of thin composite structures [37]. The physical background of this limitation is related to the length of the wave packet transmitted into the material by the PA transducer. When the distance between the echoes in the analysed A-scan is comparable or less than the width of the wave packet, the separation of these echoes becomes a challenging task for the operator or automation of defect detection. The problem can be illustrated by the following examples. Let us consider a pristine case, characterized by the A-scan shown in Figure 1a, where the front surface and back wall echoes are clearly separated. In the presence of a defect, with a large distance between the front surface and the back wall echoes (comparable or greater than the width of the wave packet), we can make a clear distinction between all echoes, i.e., localize each echo correctly. However, when echoes are partial overlapping, for example as it is shown in Figure 1b, there is overlap between the defect echo and the back wall echo and proper defect localization is either not possible or it will have a significant error when performed by conventional gating methods. Because of this phenomenon, the depth of defects that are close to the front or back walls is difficult to estimate. In addition, even when there is no echo overlap, due to the limited time resolution of A-Scan signals, the defects in C-Scan obtained from unprocessed A-Scans may appear to be much larger than the actual defects. In order to solve this problem, time resolution of A-Scan signals needs to be improved.

### 1.2. Necessity of High Frequency 10 MHz PA Transducer

There are fundamentally two resolutions in two different scanning axes which determine the imaging performance of a PA C-scan image. One is the lateral resolution, and another is the axial resolution. Furthermore, for thin composite structures, near surface resolution is also considered as an important factor in PA transducer selection for estimating the defect depth accurately. Near surface resolution depends on the extent of the dead zone of the transducer which in turn depends on the pulse-width. The lateral resolution of the transmitted ultrasound beam depends upon the centre frequency, bandwidth, and active aperture, near field distance from the transducer, material thickness and material attenuation properties. Having a larger aperture and higher frequencies results in a smaller beam width, which in turn causes a higher lateral resolution and sharper image [38,39]. The axial resolution is directly affected by the excitation frequency of PA transducers and the pulse width. For example, given the velocity of longitudinal ultrasonic wave of 3000 m/s in a specimen made of carbon fiber reinforced polymer (CFRP) and using 5, 10, and 15 MHz excitation frequencies, the wavelength of longitudinal waves are 0.6, 0.3 and 0.2 mm, respectively. For a layer thickness of 0.18 mm, we have an axial (depth) resolution for the above frequencies in C-scan approximately equal to the thickness of 3, 2, and 1 layers, respectively. We have observed that, with 5 MHz frequency, we were able to detect and accurately quantify the lateral extent of embedded inserts and impact damages in thin composites [37]. However, we were not able to resolve individual layers or plies at near surface or far surface for such a frequency, without signal processing (because the excitation pulse contains 2 to 3 cycles). Hence, a PA transducer frequency of at least 10 MHz is needed for defect depth estimation in thin composites.

However, increasing the frequency of PA transducer results in the increase of the ultrasonic attenuation and scattering noise from the material. The scattering noise is due to wavelength being much closer to ply thickness, which causes internal reflections at each resin-ply interface and results in a train of continuous noise-like signatures between the front wall and back wall of the specimen [40]. This noise is known as *structural noise* and has the same spectral characteristic as the defect echoes [41,42], and adds coherently as the ultrasound propagates in the material. Therefore, it is necessary to improve the performance of the echo localization algorithm [37] in the presence of noise by dedicated signal processing.

Another concern is about the interpretation of the PA data. Although manual interpretation is the de facto standard for composite inspections, it is time-consuming, expensive, and represents the major bottleneck of a PA inspection process. Advances in PA, full waveform data acquisition and ease of application of signal processing during post-processing, have led to a new area of research in semi-automated defect detection. Using this approach vast amount of data collected can be processed efficiently using dedicated signal processing algorithms with only the most important information presented to the NDT inspector. Additionally, 3D visualisation of defect in composite structures can lead to better defect characterisation. One further challenge of post-processing PA ultrasonic data are that the acquired phased array data of a larger area is presented by multiple strips of data. In the case of manual phased array inspection, an experienced technician spends significant amount of time for merging, aligning, stitching and adjustment of the individual strips before displaying a C-scan image of the large structure. In manual inspection, re-scanning is sometimes required, due to lack of couplant, variation of load applied on the probe or not capturing data along a straight line. However, scanning the structure by a manipulator or robot is less prone to these problems since required pressure on the wedge and uniform distribution of couplant is ensured. Thus, post processing of PA data can help to automate or eliminate some of the manual post processing needs.

In this paper, we propose an algorithm based on wavelet transform for detection and localization of defect echoes during the inspection of composite components using the high frequency ultrasonic phased array. The proposed solution offers improved axial resolution while maintaining sensible performance. We analyse two sets of scan data using 5 MHz and 10 MHz PA transducers, and address the benefit of using higher frequency ultrasonic PA transducers. Subsequently, the signal processing challenges of this choice is presented. Then, we propose solutions for the challenges and smooth the way toward semi-automated PA inspection of composite structures at high frequency 10 MHz. Finally, the paper is concluded in Section 4. The remainder of the paper is organized as follows. Section 2 begins with a short review of our echo localization algorithm with 5 MHz PA transducer presented in [37], followed by the necessity and signal processing challenges for the use of high frequency 10 MHz PA transducers. Then, a modified version of our algorithm is proposed for 10 MHz. Next, Section 3 illustrates how the algorithm is capable of visualizing echo location and post-processing results in three-dimensional plots, which can be very helpful and easy to understand by the inspector.

## 2. Echo Detection and Localization

### 2.1. Composite Material and Phased Array Configuration

The material used for the experiment consists of a carbon reinforced epoxy resin laminate of size 200 × 200 mm which is manufactured through a hand layup process using cross-ply (0/90)_12_ as stacking sequence with uni-directional pre-preg IMS-977-2-34-24KIMS-196 material (with the following material properties: $E_{1}=125$ GPa, $E_{2}=8.68$ GPa, $G_{12}=4.7$ GPa) from CYCOM and cured in an autoclave. The phased array module for data acquisition consisted of Sonatest (16:64) VEO+ series. A Sonatest PA wheel probe 5 MHz with 64 elements and 0.8 mm pitch along with a longitudinal wave 10 MHz 64 element linear phased array transducer with a pitch of 0.6 mm from Sonatest X3 series with a custom developed flat elastomer wedge, are used to acquire data at two different frequencies. Electronic scanning is performed with a group of eight active elements as active aperture, and full waveform data are acquired at 125 MHz sampling frequency during NDT inspection. The total number of A-scans that can be acquired with one line-scan of a PA transducer is equal to *M* = (number of elements – active sub-aperture +1). Therefore, for a 64-element transducer and choosing 8 elements as an active sub-aperture, the total number of A-scans will be M=64−8+1=57.

### 2.2. Baseline Echo Localization Algorithm

**Figure 2:**
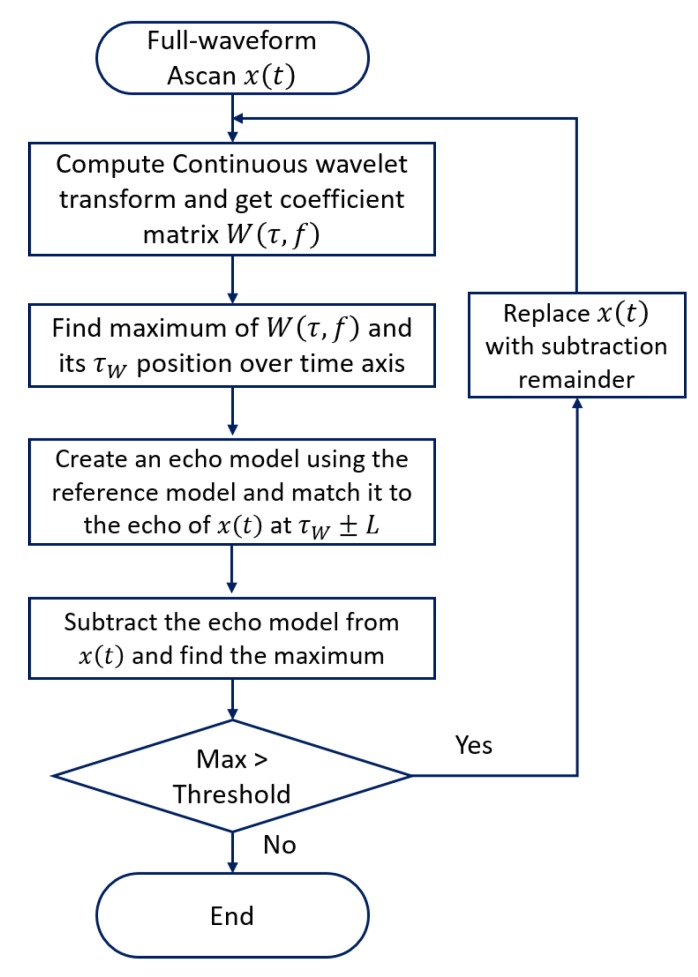
Flowchart of our baseline echo localization algorithm.

The baseline echo localization algorithm extracts the peak information for all echoes, following the stages according to the flowchart shown in Figure 2 was initially presented in [37]. It builds a *reference echo model* based on back wall echoes from a scanned area with no defects (later in the text, we may call it *reference model*). Then, with the help of wavelet transform, it searches for all echoes having an absolute amplitude above a certain threshold. Next, it finds the actual phase information of the echo and calculates exact location of the defect by analysing the phase information from the defect echo and the reference echo model. For the threshold, we use the following definition:

(1) $$\mathrm{Threshold}=\frac{\alpha }{N}\sum_{n=1}^{N}\left|x(n)\right|,$$

where α is a scaling parameter to control the threshold and *N* is the total number of samples of the original signal x(n).

Having the echo reference model, the algorithm operates as follows:

- Compute the complex continuous wavelet transform of the signal x(t) to obtain the wavelet coefficients - scalogram W(τ,f);
- The maximum value in the scalogram W(τ,f) and its $\tau_{W}$ position is located over time axis;
- An echo estimate is created by scaling the reference echo model and shifting to $\tau_{W}\pm L$ (for a small L) to find the best possible approximate of the echo of x(t). In other words, we have a window with a width of 2L+1.
- The echo estimate is removed from the original signal and the maximum amplitude (absolute) value within the residue (remainder signal) is found and compared to the threshold. If this is larger than the threshold, the resulting signal will become the input of the next iteration until the maximum value of the residue is smaller than the threshold.

**Figure 3:**
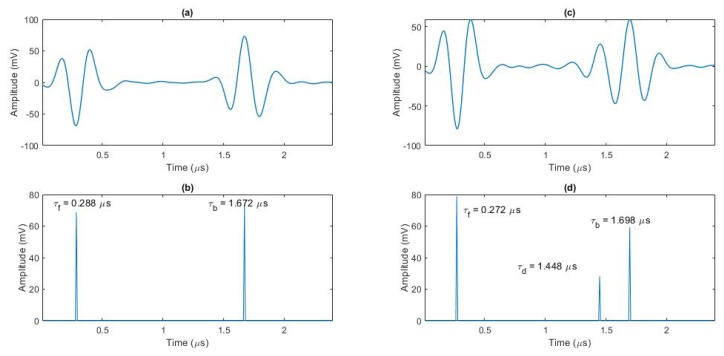
Two different A-scans using 5 MHz PA transducer: (**a**) one at non-defective location, and (**c**) an A-scan at a location with defect close to back wall; localisation results of the baseline localisation algorithm for (**a**,**c**) are shown in (**b**,**d**) respectively, providing time-of-flight for the front wall ($\tau_{f}$), defect ($\tau_{d}$) and back wall ($\tau_{b}$) echoes.

The above algorithm offers the following benefits:

- *All echoes with an amplitude above the defined threshold are detected and localized considering their phase information.* In comparison, the conventional C-scan image generation method used only one maximum peak using an absolute value of the rectified signal in the defined gate. In the best case, this gate covers the whole distance between front wall and back wall echoes. When multiple echoes are present in this gate, manual processing extracts only the strongest echo, other echoes are missed. Furthermore, phase information of the strongest echo is missed.
- *Overlapped echoes are also localized by the algorithm*. Figure 3 shows an example of using our algorithm for data obtained with 5 MHz PA transducer and its capability of localizing echoes, even partially overlapped ones. Two cases of (i) having well separated echoes and (ii) echoes with overlap are shown in Figure 3a,c. Comparing the results in Figure 3b,d, we can find the relative depths of inserts by measuring the time of flight for the front wall ($\tau_{f}$), defect ($\tau_{d}$) and back wall ($\tau_{b}$) echoes, and knowing the velocity of sound in the material. For example, assuming the ultrasonic wave velocity is 3000 m/s, the thickness of the test specimen in the areas with no inserts from Figure 3b, can be calculated as
  (2) $$x=v(\tau_{b}-\tau_{f})/2=2.1\;\mathrm{mm}.$$
  The defect depth can be obtained by using $\tau_{d}$ instead of $\tau_{b}$ and it equals to 1.8 mm for the sample with insert in Figure 3d. Having the insert thickness less than 0.1 mm, and each layer thickness of 0.183 mm, and knowing that each insert is placed between layer 10 and 11 (specimen consists of 12 layers), the above measures are reasonable.

In order to provide a clearer view for the inspector on the defect profile over the material depth, visualization of the localization results has been implemented. Section 3 describes this part in more detail.

### 2.3. Processing Data from the 10 MHz PA Transducer

The use of 10 MHz ultrasonic transducers promises higher resolution of defect (depth) detection, which will be useful in thin skin-stiffer panel and step-repaired panel to separate the echoes and characterize damages.

It was found, however, that post-processing of inspection data using 10 MHz transducer is more challenging in comparison with inspection data at 5 MHz. This is mostly due to structural noise, affected also by higher sensitivity to distribution of couplant (sprayed water) and surface roughness. A previously proposed algorithm was not performing well for this data from transducer with 10 MHz and required some modifications to address the above issues.

**Figure 4:**
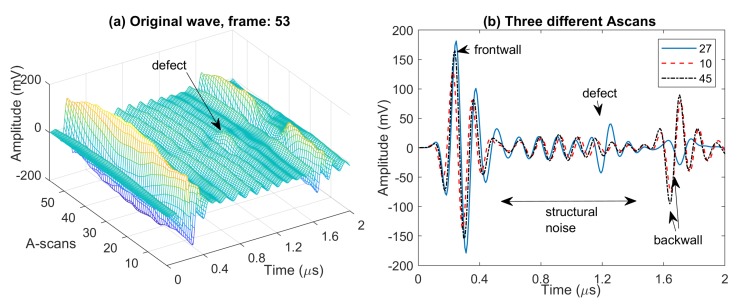
(**a**) 3D plot of full waveform scan data using 10 MHz PA transducers for a frame (one line-scan) having defect echoes, and (**b**) three selected A-scans from this frame.

Figure 4 illustrates some of the challenges for automated defect characterization with ultrasonic scanning at 10 MHz. Figure 4a shows 3D plot of a frame of scan data, and Figure 4b presents three different A-scans from the above set. We note that one frame corresponds to a single linear scan of the PA probe over the specimen. Each frame consists of 57 A-scans in our PA device set up, and B-scan can be provided as a two-dimensional image of each frame information. We use longitudinal full waveform (A-scans) data captured by a portable linear PA transducer having 64 elements with 0.8 mm pitch. In comparison to 5 MHz scan data (an example shown in Figure 3), we observe the following:

- The first challenge is the existence of multiple echoes between front wall and back wall, i.e., structural noise, corresponding to the boundaries of layers. Due to this noise, detection of defect echoes is much harder compared to the 5 MHz case, and we can only detect echoes stronger than the structural noise (i.e., having higher amplitudes).
- Another issue is the higher attenuation of the propagating wave at a higher frequency. As a result, defect echoes closer to the back wall have lower amplitudes. One practical solution found in the literature is to apply a time gain compensation that increases signal amplification with depth.
- With the 10 MHz PA transducer, the amount of couplant (water) should be as small as possible and it should be evenly distributed. Furthermore, a constant pressure should be applied to the wedge. Excess of couplant causes reverberation of the signals and increases the front wall echo length and hence the dead zone.

**Figure 5:**
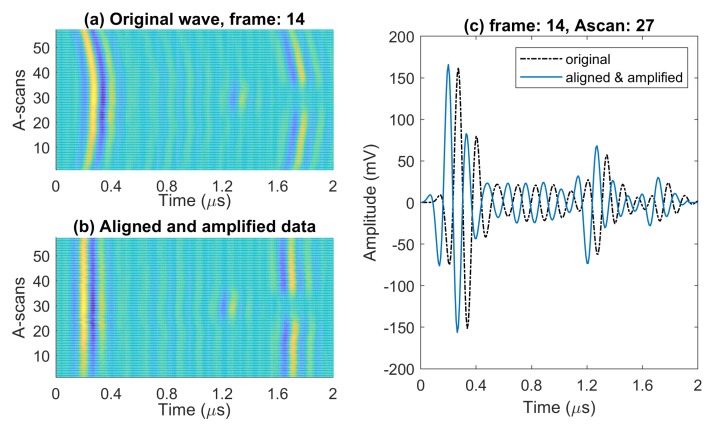
Post processing of a frame (with defect echoes) at 10 MHz: (**a**) before, and (**b**) after applying initial processing techniques (alignment, and linear slope gain), and (**c**) A-scan from defective region before and after alignment and amplification.

Due to increased sensitivity to a surface roughness at 10 MHz, we can see from Figure 5a that misalignment of A-scans is amplified in comparison to 5 MHz results. Therefore, the error in depth estimation is amplified too.

To address this issue in our signal processing, prior to the echo localization algorithm, all A-scans are aligned in time domain and amplified using time gain compensation (0.85 to 0.95 dB per mm). This is to compensate the higher attenuation we observe in 10 MHz ultrasonic wave. Figure 5b,c illustrate the alignment and amplification, respectively, for all A-scans of a frame that include defect echoes. As a result, the extracted depth information for defect echoes is more consistent.

### 2.4. Modified Echo Localization Algorithm

In this section, we present a modified echo localization algorithm that is better suited to work with 10 MHz ultrasonic data. It is worth highlighting that, prior to our echo localization algorithm, we use data pre-processing steps (namely the alignment and time-gain-compensation of the original data). Successively, the output data are fed to the localization algorithm for further processing. The following changes have been implemented in the modified algorithm:

- A threshold to ignore structural noise,
- Search Window width,
- Better echo-fit search methodology.

The algorithm searches for best echo fit is changed as follows. First, the position of the strongest echo is found from the wavelet, similar to the baseline algorithm. Then, we define a window over the max position with a width that corresponds to envelope width where its values are above the threshold. Next, we use cross-correlation between the reference echo model and the windowed signal followed by search for a combination of strongest echoes. These echoes are removed from the signal resulting in the lowest residual signal in this window. After that, the algorithm analyses the rest of A-scan signal (called residue) that has a maximum of its envelope above the threshold.

**Figure 6:**
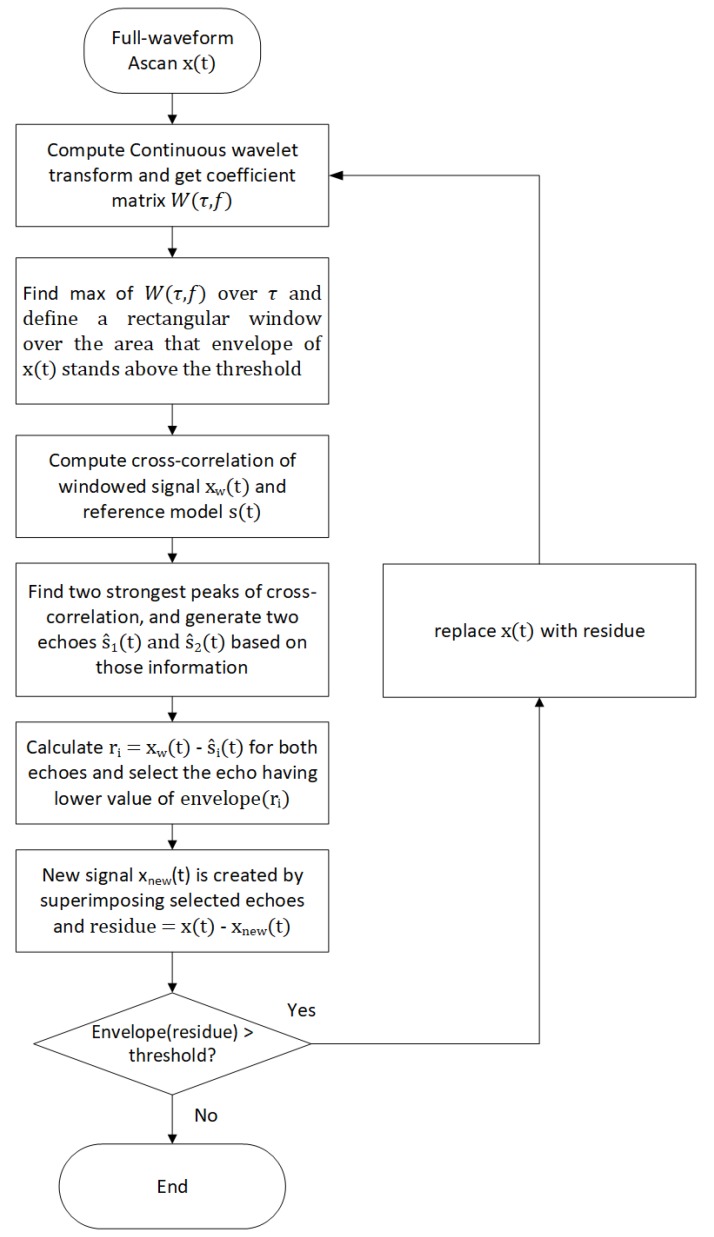
Flowchart of the modified echo localization algorithm. We note that the reference echo model and the threshold are provided as input for this algorithm.

Flowchart of the modified algorithm is presented in Figure 6. We describe these changes and modification with more detail in what follows:

1. Calculate wavelet transform of the signal,
2. Find the location of strongest peak from the wavelet scalogram,
3. Define a window at the location of the peak showing the area where envelope is above the threshold,
4. Calculate cross-correlation of the windowed signal $x_{W}\left(t\right)$ and the reference signal s(t), find the two strongest peaks with correlation coefficients $a_{1},a_{2}$ and their lag information $\tau_{1}$ and $\tau_{2}$ for the next step. It is worth noting that in general we may need superimposition of more than two echoes to model the detected defect echo. However, for our measured data, we found that two echoes corresponding to the first two peaks of the cross-correlation are sufficient for echo representation.
5. Define two echo models for the signal from the cross-correlation peaks information by applying coefficients and time-lags to the reference model, i.e., ${\hat{s}}_{i}\left(t\right)=a_{i}s\left(t-\tau_{i}\right)$ for i=1,2,
6. Subtract ${\hat{s}}_{1}\left(t\right)$ and ${\hat{s}}_{2}\left(t\right)$ from the windowed signal and select the echo model that gives us lower residue value,
7. The procedure in steps 1–6 is iteratively applied for all remaining peaks above the threshold.

**Figure 7:**
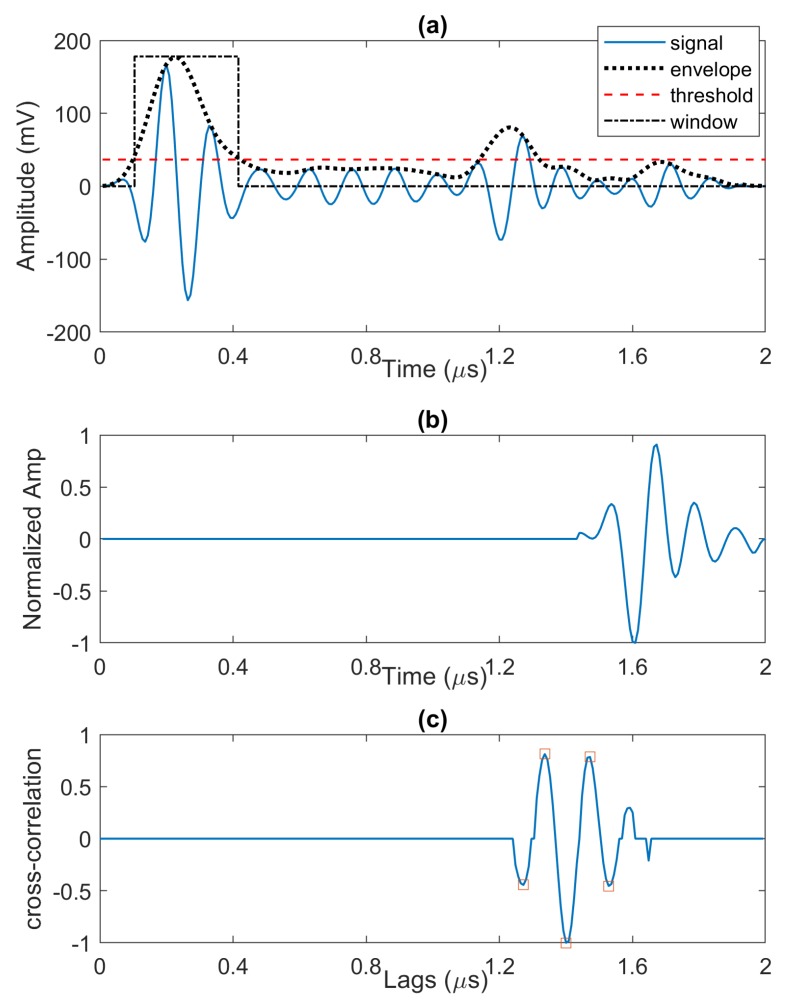
(**a**) Full wave A-scan with its envelope, threshold, and a window showing the area where envelope is above the threshold, (**b**) reference echo model, and (**c**) cross-correlation between the windowed signal and the reference model. A few largest peaks (based on absolute value of cross-correlation) are shown with red markers.

**Figure 8:**
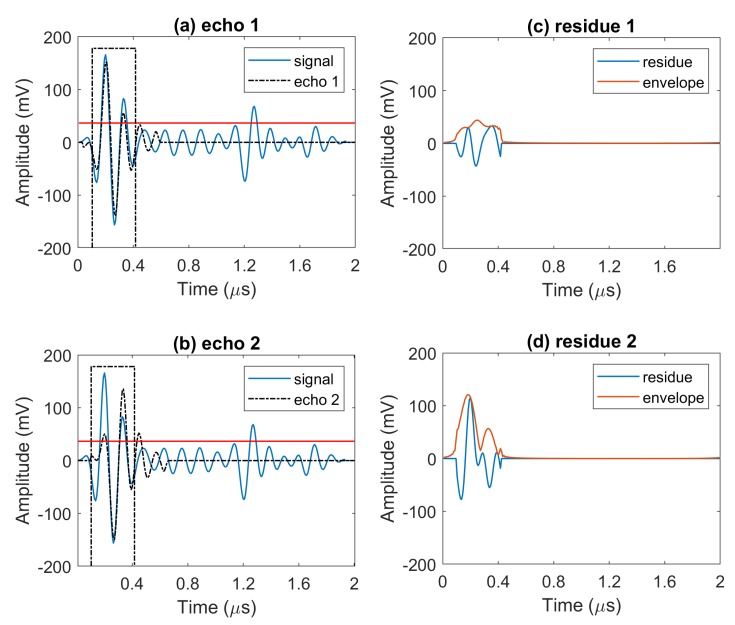
Full wave A-scan with two possible choices of echo models that differ by location, amplitude and phase in (**a**,**b**), and corresponding residue signals after subtracting echo models from the signal in (**c**,**d**).

**Figure 9:**
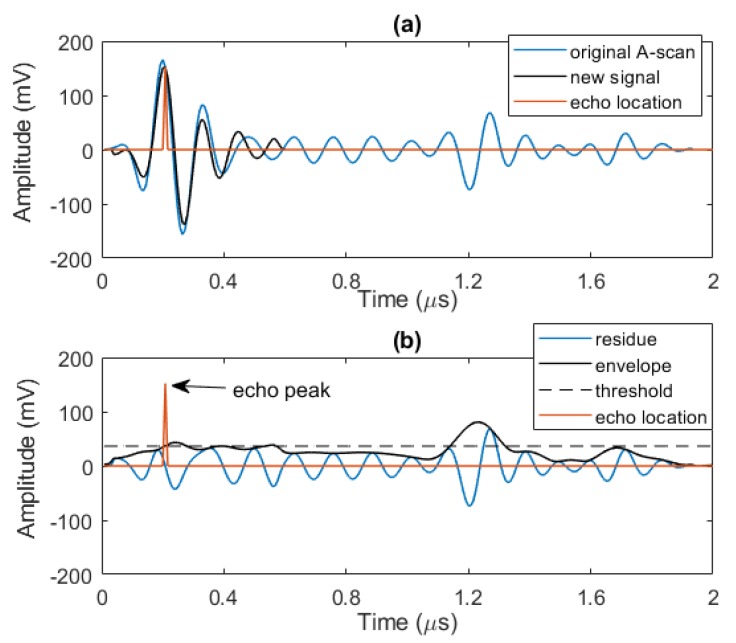
(**a**) Original A-scan and its approximate (new signal) after the 1st step of performing echo localization, (**b**) residue of the 1st step and its envelope that feeds back to the algorithm for next step, and the peak echo information.

We note that the reference echo model is a normalized version of the back wall echo that is obtained by averaging over multiple back wall echoes taken from a scan area with no defect. Figure 7, Figure 8 and Figure 9 illustrate the above steps of the algorithm.

This procedure continues iteratively until the peak echo amplitude values after subtraction in the analysis window fall below the threshold. This is presented in Figure 8 and we see that, between the two models, echo model 1 will be selected because it produces the lowest residual signal. As depicted in Figure 9, after completing the echo localization in the first window, we have a new signal showing detected echoes of the signal (solid black line), and one signal consists of extracted echoes’ peaks information (peak value as a positive or negative real number, and its location is time) plotted in solid red line. As seen in Figure 9b, the residual signal is updated and if it has a peak above the threshold, the algorithm starts again to localize remaining echoes.

An important factor here is the right value of the threshold. If it is too low, we will see wrong peaks detected due to having structural noise above the threshold. On the other side, if we select a too high threshold, we may miss some defect echoes.

### 2.5. Smart Thresholding

In order to optimise the detection process, we are looking for a solution that gives us a threshold that is adaptive with respect to the structural noise level to avoid being wrongly detected as defect echoes. We note that the structural noise should have the same shape as front wall and back wall echoes except having lower amplitudes.

In this study, we select threshold following a data driven strategy. To realise this approach, we analyze the distribution of the structural noise of the A-scans in the part of material with minimal influence from the defect-related echoes. This allows for effective threshold selection due to various conditions imposed by different amplitudes of the wave packet propagating in the media and variations in the structure of the inspected material.

**Figure 10:**
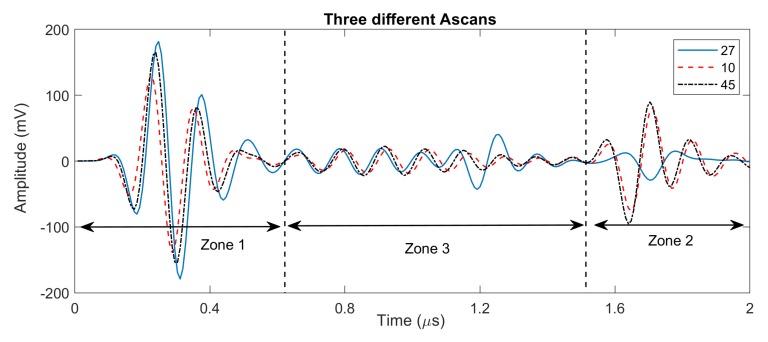
Zones 1, 2 and 3 for three different A-scans with and without defects.

**Figure 11:**
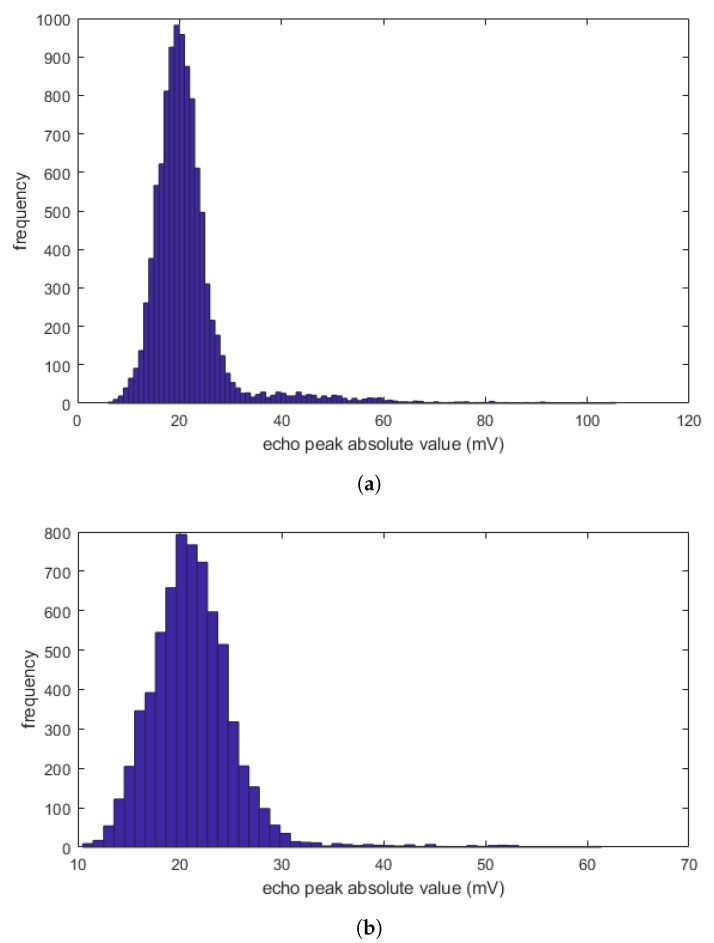
Histogram of the absolute peak values in Zone 3 for (**a**) all A-scans, and (**b**) A-scans with no defects.

In order to analyze the distribution of peak absolute value amplitudes of the structural noise, each A-scan is divided into three zones (see Figure 10): (i) Zone 1 or the dead zone that includes front wall echo, (ii) Zone 2 that only include back wall echo, and (iii) Zone 3 or the area between Zone 1 and Zone 2, and it contains structural noise and any possible defects. It is worth noting that these zones can be defined by having the prior knowledge of the dead zone and the thickness of the inspected composite material. Whenever there is any defect in Zone 3, the amplitude of front wall and/or back wall in Zones 1 and 2 will be affected. Analysing the amplitudes in Zones 1 and 2, we have been able to remove A-scans that include any defect in Zone 3 and obtain the distribution of structural noise with minimal influence from the defect echoes. Figure 11 shows an example of applying such procedure for a calibration sample (we analyse the results in more details in the next section). Comparing those two histograms, we realize that the structural noise has a distribution on the left-hand side of the histogram (with a peak approximately at 20 mV), with longer right tail as some of the few defect echoes are still remaining in the search domain.

Observing the shape of the distribution in Figure 11a,b we can assume that the underlying distribution is close to Gaussian. Therefore, to remove the anomalous right-tail of the structural noise distribution, we have decomposed the empirical distribution of the noise into the mixture of two Gaussian distributions. The decomposed distribution with lower mean is believed to be a close representation of the true structural noise distribution in an area with no defects. The mean value (μ) and standard deviation (σ) of this distribution are used to define the smart threshold. For example, we use (μ+4σ) as the smart threshold in the modified echo localization algorithm.

## 3. Three-Dimensional (3D) Visualization

Our algorithm extracts depth information of defects (based on time of flight information) for all A-scans and visualize the results in 3D plot. It is also capable of slicing the depth information into several zones and customizing each slice size which can be set, conveniently, equal to thickness of the composite layer. Detection is performed in each slice separately, for example, based on amplitude information. The depth of the zone is calculated according to time of flight information and a 3D plot containing detected defects from each zone (slice) is created. Using this technique, it is possible to quantify the defects layer-by-layer.

**Figure 12:**
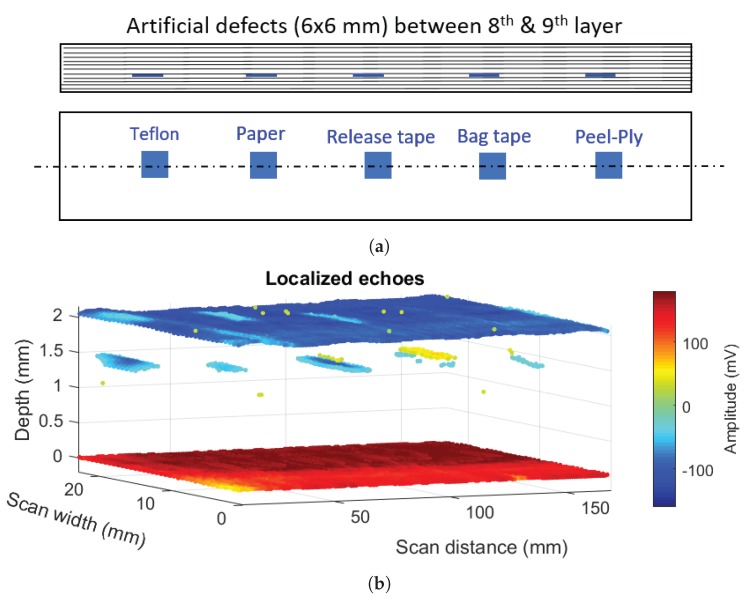
(**a**) Schematic of inserts (artificial defects) in the calibration sample and (**b**) 3D visualization of localized echoes using our modified algorithm and the smart thresholding.

Figure 12 shows the result of using our signal processing, on the 10 MHz scan data in the calibration sample with 12 layers and different types of inserts of size 6 × 6 mm located between 8th and 9th layers of the specimen, i.e., at a depth of eight layers [37]. Figure 12a shows a schematic diagram of the inserts between 8th and 9th layers of the calibration sample with the following material types from left to right: Teflon, Paper, Release tape, Bag tape, and Peel ply. Figure 12b shows a volumetric plot of all echoes (with an amplitude above the threshold) versus thickness. It includes echoes from front wall, back wall and defects. The colour map shows the amplitude of the echo at each point considering the actual amplitude information from the full waveform A-scan signal (i.e., it can be either positive or negative). Front wall peak position is taken as the reference for depth measurement.

**Figure 13:**
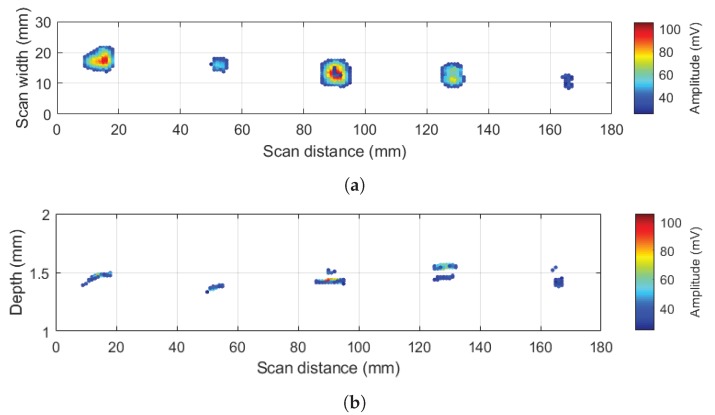
(**a**) sizing, and (**b**) depth information of 6x6 mm inserts in the calibration sample.

Figure 13a,b plots the size and depth information of defects. Size and depth estimates of these artificial defects (6 × 6 mm inserts) are shown in Table 1 in more details. Sizing is performed by a part of our code and it calculates the surface of detected defects in Figure 13b in mm${}^{2}$. We see that estimated depth is quite accurate. Regarding the sizing at the first look, we see that some inserts are oversized. One can notice that different materials have different reflectivity. For example, Teflon and Release tape inserts have stronger reflections compared to other types. This effect is reflected in Table 1, where size estimation for these two types are larger compared with others. Furthermore, Figure 13a shows that outer borders of detected defects have lower amplitude compared to the inside areas.

**Figure 14:**
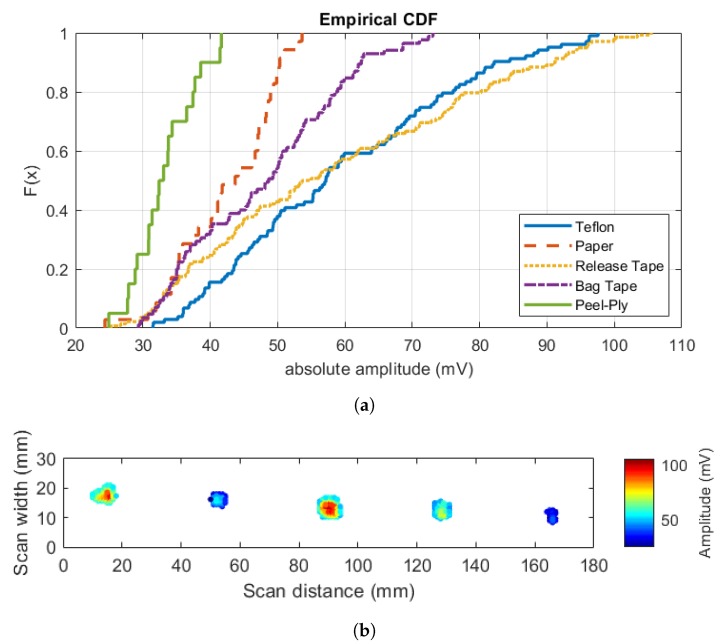
(**a**) Empirical CDF of absolute peak of defect echoes, and (**b**) refined size estimation after resizing defects with a wide range of amplitude spread.

We believe that the observed error in detected defect sizes is due to different reflection properties of different insert materials. We address this issue by removing a lower 40th percentile of the cumulative distribution function (CDF) of absolute peak values of detected defect echoes shown in Figure 14a. Results of such procedure are shown in Figure 14b. For this case, we achieve following defect sizes (in mm${}^{2}$): Teflon (reduced to 36.6), Paper (no change, 21), Release tape (reduced to 44.4), Bag tape (reduced to 30.6), and Peel-ply (no change, 12).

## 4. Conclusions

In this paper, we analyzed signal processing challenges of NDT of aerospace grade composite using an ultrasonic PA module in an autonomous inspection platform designed for the EU-H2020 FET-OPEN CompInnova project. The post processing of the PA data with the proposed algorithm leads to resolving overlapped echoes and improving defect detection and axial (depth) resolution. We demonstrated an approach that allows achieving good results at low hardware and computational costs. We addressed the signal processing challenges for the use of high frequency 10 MHz PA transducers, and proposed solutions to overcome these problems. The proposed hardware solution is relatively inexpensive due to a conservative selection of the frequency (10 MHz), and it is going to be deployed using the robotic platform developed within the CompInnova project. This solution allows for eliminating some of the adverse effects of manual scanning related to non-uniform pressure distribution and couplant application, and increased repeatability of the scanning process.

The proposed modification of the resolution enhancement algorithm allows for robust detection of the defects close to either front or back walls of the inspected structure and resolution improvement up to the ply thickness of composite material while remaining simple in implementation. The algorithm also benefits from the smart thresholding technique that allows for keeping good performance in terms of peaks due to structural noise overlapping with defect echoes, while keeping defect detection sensitivity at or above the level of conventional immersion-based ultrasonic scanning. Another advantage of the proposed approach is intuitive 3D visualization of the inspected material volume that is highly suitable for semi-automated data analysis and interpretation, where routine post-processing tasks are automated and an inspector is only evaluating results presented in easy to perceive 3D form.

## Figures and Tables

**Table 1:** Size and depth estimation of different types of defects with the size (6×6 mm) at the design depth 1.47 mm in the reference standard specimen.

	Teflon	Paper	Release Tape	Bag Tape	Peel Ply
Automated defect depth (deepest) estimate (mm)	1.50	1.39	1.52	1.57	1.54
Automated defect depth estimate error (%)	2%	5.4%	3.4%	6.8%	4.8%
C-scan Manual defect size estimate (mm${}^{2}$)	30	9	38.4	faintly detected	8.4
Automated defect size estimate (mm${}^{2}$)	53.4	21	59.4	43.2	12
Automated defect size estimate error (%)	43.8%	−41.7%	65%	20%	−66.7%
Manual defect size estimate error (%)	−17%	−75%	7%	not applicable	−77%

## Abbreviations

The following abbreviations are used in this manuscript:

AR	Auto-Regressive
CDF	Cumulative Distribution Function
CFRP	Carbon Fiber Reinforced Polymer
IRT	Infrared Thermography
NDT	Non-Destructive Testing
PA	Phased Array
SNR	Signal-to-Noise-Ratio
ToF	Time of Flight
